# The DExH Box Helicase Domain of Spindle-E Is Necessary for Retrotransposon Silencing and Axial Patterning During *Drosophila* Oogenesis

**DOI:** 10.1534/g3.114.014332

**Published:** 2014-09-19

**Authors:** Kristen M. Ott, Tram Nguyen, Caryn Navarro

**Affiliations:** * Department of Medicine, Biomedical Genetics, Boston University School of Medicine, Boston, Massachusetts 02118; † Graduate Program in Genetics and Genomics, Boston University School of Medicine, Boston, Massachusetts 02118

**Keywords:** piRNA, *Drosophila*, retrotransposon, oogenesis, embryonic patterning, RNA helicase

## Abstract

Transposable selfish genetic elements have the potential to cause debilitating mutations as they replicate and reinsert within the genome. Therefore, it is critical to keep the cellular levels of these elements low. This is especially true in the germline where these mutations could affect the viability of the next generation. A class of small noncoding RNAs, the Piwi-associated RNAs, is responsible for silencing transposable elements in the germline of most organisms. Several proteins have been identified as playing essential roles in piRNA generation and transposon silencing. However, for the most part their function in piRNA generation is currently unknown. One of these proteins is the *Drosophila melanogaster* DExH box/Tudor domain protein Spindle-E, whose activity is necessary for the generation of most germline piRNAs. In this study we molecularly and phenotypically characterized 14 previously identified *spindle-E* alleles. Of the alleles that express detectable Spindle-E protein, we found that five had mutations in the DExH box domain. Additionally, we found that processes that depend on piRNA function, including Aubergine localization, Dynein motor movement, and retrotransposon silencing, were severely disrupted in alleles with DExH box domain mutations. The phenotype of many of these alleles is as severe as the strongest *spindle-E* phenotype, whereas alleles with mutations in other regions of Spindle-E did not affect these processes as much. From these data we conclude that the DExH box domain of Spindle-E is necessary for its function in the piRNA pathway and retrotransposon silencing.

A large portion of both the human and Drosophila genomes are composed of transposable elements (TEs), which are capable of creating genome instability and a high mutation rate on excision and re-integration within the genome (Belgnaoui *et al.* 2006; Gasior *et al.* 2006). In most organisms germ cells seem to be particularly sensitive to elevated levels of TEs, and TE deregulation ultimately leads to germ cell developmental defects and sterility (Juliano *et al.* 2011). TE regulation in the germline is particularly important as germline DNA is inherited by offspring and mutations can hinder reproductive success or could be deleterious to the progeny.

The *Drosophila* ovary is composed of both somatic and germ cells, and in both cell types a highly conserved class of small noncoding RNAs, piRNAs (Piwi-interacting RNAs), are responsible for silencing TE expression and transposition (Guzzardo *et al.* 2013). Germline piRNAs are highly abundant and quite divergent in their sequences. Although the population of piRNAs is quite complex, most piRNAs can be mapped to a small number of genomic regions called “piRNA clusters” (Brennecke *et al.* 2007). Precursor piRNAs (Pre-piRNAs) are transcribed as long single-stranded RNAs from these clusters. Pre-piRNA transcripts are exported from the nucleus and processed into primary piRNAs. In germ cells, transcription is controlled by several chromatin-associated proteins, including the HP1 paralog Rhino and its binding partner Cutoff (CUFF), the histone methyltransferase, dSETDB1, as well as the Tudor domain proteins, Kumo/Qin and Vreteno (VRET) (Anand and Kai 2012; Handler *et al.* 2011; Klattenhoff *et al.* 2009; Pane *et al.* 2011; Rangan *et al.* 2011; Zamparini *et al.* 2011; Zhang *et al.* 2011). Primary transcripts are bound by the putative helicase, UAP56, and shuttled out of the nucleus, where they are transferred to Vasa (VAS) within a specialized perinuclear cytoplasmic region known as the nuage (Lim and Kai 2007; Zhang *et al.* 2012). The nuage is believed to be the site of retrotransposon silencing (Lim and Kai 2007). These long transcripts are then processed further to mature primary piRNAs. The 5′ end of the mature primary piRNA is likely generated by the endonuclease Zucchini (Ipsaro *et al.* 2012; Nishimasu *et al.* 2012; Voigt *et al.* 2012). However, the complete mechanism by which the mature piRNAs are generated is currently unknown. Several other proteins have been identified as necessary to generate primary piRNAs, most localize to the nuage, and several form complexes; however, how many of these proteins function in piRNA biogenesis is not known (Czech *et al.* 2013; Guzzardo *et al.* 2013; Handler *et al.* 2013).

In germ cells, cytoplasmic primary piRNAs also enter into an amplification cycle (Brennecke *et al.* 2007; Gunawardane *et al.* 2007). Here, proteins of the Argonaute family bind piRNAs. In *Drosophila*, these include Piwi and Aubergine (Aub) (Brennecke *et al.* 2007; Gunawardane *et al.* 2007). It is unclear what role Piwi plays in germline piRNA generation. Deep sequencing of piRNAs bound by Aub has shown that it binds piRNAs that are mostly antisense to active TE mRNAs. Active TE mRNAs are cleaved 10 nucleotides downstream of the piRNA terminal A, most likely through AUB’s slicer activity, thereby generating secondary sense piRNAs (Brennecke *et al.* 2007; Gunawardane *et al.* 2007). Sense piRNAs are loaded onto another Argonaute family protein Argonaute 3 (Ago3), which functions to cleave cluster-derived antisense transcripts to generate more antisense piRNAs. This mechanism of piRNA generation has been termed the “ping-pong” amplification cycle and provides an adaptive response to the presence of newly synthesized TE mRNA. This amplification cycle most likely takes place in the nuage (Lim and Kai 2007). Most proteins necessary for piRNA biogenesis localize to the nuage and a temporal hierarchical relationship governing nuage localization exists among these proteins. Vasa, an RNA helicase, localizes first, followed by the DExH box helicase/Tudor domain protein, Spindle-E (SPN-E), and the Tudor domain protein, Tejas (TEJ), both of which are dependent on VAS for their localization (Findley *et al.* 2003; Lim and Kai 2007; Malone *et al.* 2009; Patil and Kai 2010). Other piRNA pathway proteins such as AUB, Ago3, and Krimper (KRIM) rely on VAS, SPN-E, and TEJ for their localization. The cumulative data indicate that a large complex or several complexes form at the nuage or localize piRNA proteins to the nuage, where the piRNA proteins along with their associated piRNAs act to silence retrotransposons.

How piRNAs function to silence TEs is currently not well-established, but evidence exists for both post-transcriptional, as described above, and transcriptional regulation. Recently, it has been shown that PIWI is responsible for regulating retrotransposon expression in ovarian somatic cells, as the occupancy of RNA Polymerase II at retrotransposon promoters is increased along with steady-state levels of transposon transcripts in *piwi* mutant cells (Huang *et al.* 2013; Le Thomas *et al.* 2013; Rozhkov *et al.* 2013; Sienski *et al.* 2012). It is currently unknown whether this mechanism also occurs in the germ cells. How piRNAs influence transcription is also currently unknown, but it has been reported that heterochromatin protein binding and histone methylation at retrotransposon sequences changes in piRNA pathway mutants where piRNA levels are decreased (Klenov *et al.* 2007; Pal-Bhadra *et al.* 2004).

A critical protein involved in the generation of most germ cell piRNA species is *Drosophila* Spindle-E (Malone *et al.* 2009). SPN-E colocalizes to the nuage along with other piRNA pathway proteins and its function is required for either primary piRNA generation and/or the ping-pong cycle (Malone *et al.* 2009; Patil and Kai 2010). *spn-E* was originally identified as a gene necessary for microtubule network formation, RNA localization, and embryonic pattern formation (Gillespie and Berg 1995; Klattenhoff *et al.* 2007; Martin *et al.* 2003). However, it is not known whether SPN-E function in the piRNA pathway controls all of these processes. The SPN-E protein contains a DExH box helicase domain, a Tudor domain, and a Zinc finger (Zn), which implicate its function in RNA processing, translational regulation, RNA decay, splicing, or protein–protein interactions (Figure 1, A and B). However, the relative contribution of these domains to SPN-E function, particularly in the piRNA pathway, is currently unknown. Therefore, to begin to understand how SPN-E functions during oogenesis, particularly in TE silencing, we took advantage of several previously isolated *spn-E* mutant fly lines in an attempt to identify mutations in the predicted functional domains. Our results provide evidence that the DExH box helicase domain of SPN-E is necessary for TE silencing in the germline.

**Figure 1:**
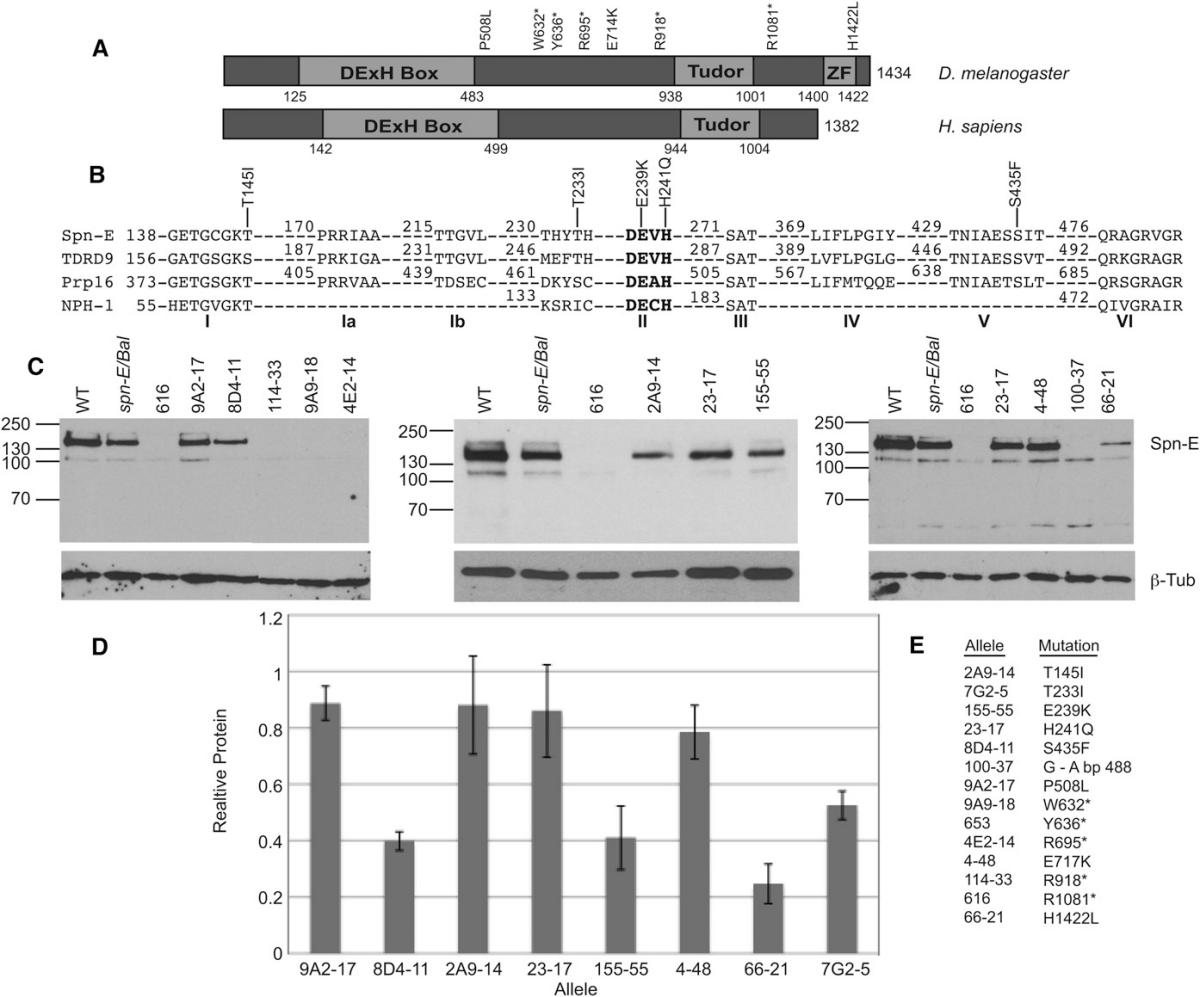
Eight of the fourteen *spn-E* alleles express detectable protein and have point mutations in the SPN-E coding region. (A) Domain structure of *Drosophila* SPN-E and its human homolog, TDRD9. SPN-E contains a highly conserved DExH box and a Tudor domain as well as a Zinc finger, whereas TDRD9 only has a DExH box and Tudor domain. The position of the two mutations outside of the conserved domains, the five mutations that do not produce detectable protein, and the Zinc finger are shown. (B) The amino acid sequence of the SPN-E DExH box domain compared with its human homolog TDRD9, yeast splicing factor Prp16, and vaccinia virus protein NPH-I. The positions of the five mutations identified in the SPN-E DExH box domain are shown. Amino acid numbering is according to Ensemble Genome Browser release 73. (C) SPN-E protein expression in mutant ovary extracts as measured by Western blotting. Protein was isolated from hemizygous ovaries of the genotype *spn-E^mutant^/spn-E^∆125^*. Eight alleles express detectable protein of the correct size for SPN-E. Four alleles do not express detectable protein. *Spn-E/Bal = spn-E^∆125^/Balancer* chromosome. Line 7G2-5 is not shown. Several extraneous bands are found on the Western blots shown above. We did not detect these bands when we used a second antibody developed in the laboratory of Dr. Toshie Kai (Patil and Kai 2010; data not shown); therefore, we think that the extra bands are most likely nonspecific bands recognized by our SPN-E antibody. (D) SPN-E protein levels in the various mutant ovaries relative to *spn-E^∆125^*/Balancer. Error bars represent SD of at least 2 separate protein isolates. SPN-E protein levels were normalized to beta-tubulin. (E) A listing of each *spn-E* allele name along with its corresponding mutation.

## Materials and Methods

### *Drosophila* strains

The following *Drosophila* lines were used: w; FRT[ry^+^]82B spn-E^4-4 8^e, w; FRT[ry^+^]82B spn-E^66-21^ e, w; FRT[ry^+^]82B spn-E^100-37^ e, w; FRT[ry^+^]82B spn-E^189-3 9^e, w; FRT[ry^+^]82B spn-E^114-33^ e, w; and FRT[ry^+^]82B spn-E^155-5 5^e, and w; FRT[ry^+^]82B spn-E^23-17^ e were a kind gift from Ruth Lehmann (Staeva-Vieira 2003). yw; FRT[ry^+^]82B spn-E^2A9-14^, yw; FRT[ry^+^]82B spn-E^9A2-17^, yw; FRT[ry^+^]82B spn-E^9A9-18^, yw; FRT[ry^+^]82B spn-E^8D4-11^, yw; and FRT[ry^+^]82B spn-E^4E2-14^, and yw; FRT[ry^+^]82B spn-E^7G2-5^ were a kind gift from Daniel St. Johnston (Martin *et al.* 2003). spn-E^∆125^ was a kind gift from Celeste Berg (Gillespie and Berg 1995). spn-E^653^ and spn-E^616^ were obtained from the Tubingen stock collection (Tearle and Nusslein-Volhard 1987). The wild-type strain used was Oregon-R.

### Induction of germline clones

Germline clones were induced using the FLP/FRT system (Chou and Perrimon 1996). *FRT82B spn-E* females were crossed to *yw hsflp(ii)*; *FRT82B UbiGFP* male flies (Bloomington stock center). Second or third instar larvae were heat-shocked at 37° for 2 hr on two consecutive days to induce clones. Female flies of the correct genotype were dissected 10 d after heat shock.

### Sequencing

Genomic DNA was isolated from 30 adult male *spn-E^mutant^/spn-E^∆125^* flies as in (Rehm). *spn-E^∆125^* is a deletion that completely removes the *spn-E* gene (Gillespie and Berg 1995). The *spn-E* gene was sequenced using a primer walking strategy. Sections of the *spn-E* gene were amplified using standard PCR conditions and Crimson Taq (New England Biolabs). Multiple sets of *spn-E* gene-specific primers that span the gene from the start codon to the stop codon, including introns, were used (primer sequences available upon request). PCR products were purified using the Qiagen MinElute PCR purification kit. Sanger sequencing was performed using the Big Dye termination kit.

### Aubergine and Spindle-E antibody production

Rabbit polyclonal antisera directed against peptide MNLPPNPVIARGRGRG (amino acids 1–16) (Brennecke *et al.* 2007) of AUB and TNHRRKHSIGKFYRDQLG (amino acids 295–312) of SPN-E were generated by Pocono Rabbit Farm and Laboratory, Inc., using their Quick Draw 49-Day protocol. The antiserum was affinity purified by Pocono Rabbit Farms using the appropriate peptide.

### Protein isolation and western blot analysis

Fifteen to 20 female flies that were 2 or 3 days old were placed on yeast overnight and ovaries were dissected in 1× Ephrussi Beadle Ringer’s buffer (EBR, 130 mM NaCl, 4.7 mM KCl, 1.9 mM CaCl_2_, and 10 mM Hepes, pH 6.9). Ovaries were homogenized five- to 10-times in lysis buffer [50 mM Tris-HCl pH 8.0, 150 mM NaCl, 1% NP40, 1× Halt protease inhibitor single-use cocktail (Thermo Scientific)]. Samples were centrifuged twice for 5 min at 12,000*g* at 4° and the supernatant was assayed for protein concentration. Protein was quantitated by a Bradford-type assay using the Biorad Protein Assay kit; 40 μg of protein was resolved on 7% SDS-polyacrylamide gels and transferred to PVDF membranes (Immobilon) using the Bio-Rad mini-PROTEAN tetra electrophoresis system. Western blots were performed as described previously (Navarro *et al.* 2004). Primary antibodies were diluted at 1:1000 in 5% nonfat milk in Tris-buffered saline with 0.5% Tween. Antibodies used were mouse anti-β-tubulin (Developmental Studies Hybridoma Bank) and affinity purified rabbit anti-Spindle-E. HRP-conjugated secondary antibodies (Jackson ImmunoResearch Laboratories) were used at a dilution of 1:10,000. For detection, the blots were incubated in Amersham ECL Prime Western Blotting Detection Reagent (GE Healthcare) according to the manufacturer’s instructions and exposed to X-ray film (Kodak Biomax light or Amersham). Bands were quantitated using NIH ImageJ software.

### D/V patterning assay

Eight to 10 female flies that were 2 or 3 days old of the specified genotypes (*spn-E^mutant^/Deficiency* or *spn-E^mutant^/Balancer)* were placed on yeast overnight, put into egg-laying chambers, and allowed to lay eggs on apple juice agar plates with yeast paste overnight at 25°. Eggs of each class were counted after 24 hr of egg-laying for three consecutive days.

### Real-time quantitative RT-PCR

Fifteen to 20 female flies (*spn-E^mutant^/Deficiency* or *spn-E^mutant^/Balancer*) that were 2 or 3 days old were placed on yeast overnight. Ovaries were dissected in EBR and placed in microcentrifuge tubes. The EBR was removed and the ovaries were flash-frozen in liquid nitrogen or used fresh for RNA isolation. RNA was isolated using TRIzol reagent (Invitrogen) according to the manufacturer’s instructions. RNA was treated twice with Turbo DNase (Ambion) to remove contaminating genomic DNA. cDNA was generated from 1 µg of RNA using the Verso cDNA kit (Thermo Scientific) or the Maxima cDNA kit (Fermentas). A 10-µl real-time PCR reaction was performed with either 1× ABsolute Blue SYBR Green master mix (Abgene), 0.075 mM of forward and reverse primers, and 1 µl of cDNA reaction or 1× Maxima SYBR master mix (Fermentas), 0.3 mM of forward and reverse primers, and 1 µl of cDNA reaction. Cycling parameters were: 50°, 2 min; 95°, 10 min; 95°, 15 sec; and 60°, 1 min for 40 cycles using an ABI 7900HT. The following previously published primers were used: HetA and TART as described elsewhere (Pane *et al.* 2007); I-Factor and roo as described elsewhere (Vagin *et al.* 2006); Blood as described elsewhere (Donertas *et al.* 2013); gypsy as described previously (Brennecke *et al.* 2007); and Adh as described previously (Klenov *et al.* 2007). Data were analyzed using SDS software and relative RNA levels were calculated by the 2^−∆∆Ct^ method (Livak and Schmittgen 2001). RNA was normalized to Adh levels. Fold enrichments were calculated in comparison with respective RNA levels obtained from heterozygous flies.

### Immunohistochemistry and microscopy

Antibody staining was performed as described previously (Navarro *et al.* 2004). Chicken anti-GFP (Abcam) was used at a dilution of 1:5000, rabbit anti-Egalitarian was used at a dilution of 1:5000 (Navarro *et al.* 2004), rabbit anti-Aubergine was used at a dilution of 1:1000, and mouse anti-Gurken clone 1D12 was obtained from the Developmental Studies Hybridoma Bank and was used at a dilution of 1:20 (Queenan *et al.* 1999). Secondary antibodies (Cy3, Jackson Immunoresearch; Alexa488, Molecular Probes) were used at a concentration of 1:500. Images were captured using a Zeiss 510 LSM confocal microscope and processed using ImageJ and/or Photoshop software.

## Results

### Molecular analysis of *spn-E* alleles

We obtained 12 *spn-E* alleles from two independent EMS mutagenesis screens and determined if each line expressed ovarian SPN-E protein (Martin *et al.* 2003; Staeva-Vieira 2003) (Figure 1, C and D). Eight alleles expressed protein of the correct size for full-length SPN-E. Four of these eight alleles (9A2-17, 2A9-14, 23-17, 4-48) expressed SPN-E protein at levels close to wild-type. Four other alleles produced protein below wild-type levels (8D4-11, 155-55, 66-21, 7G2). The 66-21 mutant ovaries have approximately 75% less SPN-E protein than wild-type ovaries. This allele displays only a mild phenotype (see below); therefore, it seems that the level of SPN-E produced by 66-21 is enough for mostly wild-type function. All of the other protein expressing *spn-E* lines produce more protein than 66-21. Therefore, we conclude that the phenotypes that we describe below are probably not due to reduced SPN-E protein levels, but rather are due to disruption of protein function by the point mutation. Four of the 12 alleles (114-33, 9A9-18, 4E2-14, and 100-37) as well as two additional *spn-E* alleles that had been previously phenotypically characterized (616 and 653) (Tearle and Nusslein-Volhard 1987) do not express detectable SPN-E protein (Figure 1C, Supporting Information, Figure S1).

We next determined the genomic DNA sequence from the translational start codon to the stop codon, including introns, for each allele to determine if the *spn-E* gene contained mutations that lie in the known functional domains: the DExH box; the Tudor; or the Zinc finger (Figure 1A). Our sequencing of the protein expressing alleles identified five mutations in the DExH box domain, one mutation in the Zinc finger domain, and two mutations in highly conserved residues outside of the predicted functional domains (Figure 1, A and B, Table 1). Additionally, all alleles that did not express detectable protein had mutations that cause premature stop codons to be formed (Figure 1, A and B Table 1).

**Table 1:** Overview of the *spn-E* mutant phenotypes

Allele Name	SPN-E Protein	Mutation	Affected Domain	D/V Defects	Retrotransposon Expression	Dynein Aggregate Formation	Aub Nuage Localization
2A9-14	+	Thr145Ile	DExH box (I)	Severe	↑↑↑	Yes	Delocalized
7G2-5	+	Thr233Ile	DExH box (I/II)	Severe	↑↑↑	Yes	Delocalized
155-55	+	Glu239Lys	DExH box (II)	Mid	↑↑↑	Yes	Delocalized
23-17	+	His241Gln	DExH box (II)	Mild	↑	No	Partial
8D4-11	+	Ser435Phe	DExH box (V)	Severe	↑↑↑	Yes	Delocalized
9A2-17	+	Pro508Leu	None	Mild	↑↑	No	Partial
4-48	+	Glu717Lys	None	Mild	↑	No	Nuage
66-21	+	His1422Leu	Zn finger	Mild	↑	No	Nuage
100-37	—	G→A bp 488 splice site	Truncated at aa 151	Mid	↑↑↑	Yes	Delocalized
114-33	—	Arg918stop	Truncated at aa 918	Severe	↑↑↑	Yes	Delocalized
9A9-18	—	Trp632stop	Truncated at aa 632	Severe	↑↑↑	Yes	Delocalized
4E2-14	—	Arg695stop	Truncated at aa 695	Severe	↑↑↑	Yes	Delocalized
616	—	Arg1081stop	Truncated at aa 1081	Mid	↑↑↑	Yes	Delocalized
653	—	Tyr636stop	Truncated at aa 636	Severe	↑↑↑	Yes	Delocalized

+ or − for SPN-E protein indicates: (+) SPN-E protein detected; (−) SPN-E protein not detected on Western blot analysis. Number in parentheses next to DExH box indicates the DExH box motif affected. Severe D/V defects indicate mostly collapsed eggs laid by mutant females; mid indicates the majority of eggs laid were collapsed; however, a small percent have wild-type or fused dorsal appendages; mild indicates the majority of eggs laid were wild-type. ↑↑↑ indicates all retrotransposons were upregulated in the mutant ovaries; ↑↑ indicates that at least two retrotransposons tested were upregulated; ↑ indicates that one or no retrotransposons was upregulated in the mutant ovaries. bp numbering for 100-37 according to Ensemble Genome Browser. ND, not determined.

DExH box helicase domains consist of several motifs, some of which have defined functions associated with helicase activity (Luking *et al.* 1998). We identified mutations in motif I (2A9-14^T145I^, the Walker A box), a common motif found in proteins that bind and hydrolyze NTPs. Motif II (155-55^E239K^, 23-17^H241Q^, the DExH region), which is necessary for ATP binding/hydrolysis and interdomain contacts, and motif V (8D4-11^S435F^), which may be necessary for RNA binding. In addition, we identified a mutation that is between motifs I and II in an amino acid that is conserved between species (7G2-5^T233I^) (Figure 1B). The identification of multiple mutations in the DExH box domain from two independent genetic screens indicates that the DExH box domain may be critical for SPN-E function.

Zn fingers are mostly known for their role in DNA binding and transcriptional regulation; however, they have also been shown to be necessary for RNA, protein, and lipid binding (Gamsjaeger *et al.* 2007; Hall 2005; Matthews and Sunde 2002). The Zn finger found in SPN-E belongs to the Cys_2_His_2_-like fold group that is identified by the sequence, X_2_-Cys-X_2,4_-Cys-X_12_-His-X_3,4,5_-His, where the two Cys and two His amino acids are important for coordinating Zn (Pabo *et al.* 2001). The mutation that we identified changes the last His in the Zn finger region to a Leu (66-21, aa 1422). Because this amino acid is important for Zn coordination, the mutation that we found most likely would disrupt the function of the domain.

Because we found mutations in two of the conserved functional domains of SPN-E, we wanted to determine if these amino acid changes affect protein function. Therefore, we assessed the extent to which the mutations affect developmental processes known to require SPN-E function such as dorsal/ventral (D/V) eggshell patterning, AUB subcellular localization, and dynein motor complex localization. Additionally, we directly tested whether the piRNA pathway was affected in the mutant ovaries by measuring retrotransposon levels (Table 1).

### DExH box domain mutations in SPN-E perturb its function in dorsal/ventral patterning

*spn-E* mutant females lay eggs with severe D/V patterning defects ranging from fused dorsal appendages to no dorsal appendages and collapsed eggs (Gillespie and Berg 1995). To evaluate the importance of the DExH box and Zn finger domains to the establishment of D/V eggshell polarity, we examined eggs laid by the various mutant females. The eggs laid by the *spn-E* mutant flies that we describe here vary in the severity of D/V patterning defects (Table 2). In contrast to wild-type females that lay eggs with two distinct dorsal appendages, most of the eggs laid by females expressing SPN-E protein with DExH box domain mutations showed a very severe phenotype similar to the strongest *spn-E* mutant egg phenotype, with a high percentage of collapsed eggs or eggs with no dorsal appendages. For example, female flies expressing SPN-E protein with a mutation in amino acid 145 (2A9-14^T145I^), which lies in DExH box motif I, lay approximately 94% collapsed eggs and no wild-type eggs. This is in contrast to wild-type females that do not lay collapsed eggs (*spn-E^∆125^*/*Balancer*), but compares favorably with eggs laid by *spn-E* mutant females that do not express detectable SPN-E protein, which lay between 83% and 100% collapsed eggs (114-33^R918*^, 9A9-18^W632*^, 4E2-14^R695*^). The same severe phenotype occurs in eggs laid by *spn-E* mutant females that have mutations located in DExH box motif V (8D4-11^S435F^) and in between motifs I and II (7G2-5^T233I^).

**Table 2:** Most *spn-E* mutant females lay eggs with dorsal/ventral patterning defects

Allele Name	% Wild-Type	% Fused	% None	% Collapsed	Total Eggs
*spn-E/Bal*	100	0	0	0	**600+**
**Alleles with mutation in DExH box**					
2A9-14	0	0.48 ± 0.078	5.9 ± 3.5	93.7 ± 3.6	**428**
7G2-5	4.3 ± 3.3	3.9 ± 1.9	3.4 ± 2.6	88.5 ± 7.8	**759**
155-55	5.7 ± 2.4	12.1 ± 2.5	18.5 ± 9.3	63.7 ± 9.5	**742**
23-17	54.7 ± 9.1	31.5 ± 2.1	10.7 ± 2.5	4.2 ± 3.3	**970**
8D4-11	0	0	0.95 ±1.3	99.1 ± 1.3	**181**
**Alleles with mutation outside of predicted domains**					
9A2-17	59.4 ± 23.5	37.9 ± 21.6	2.6 ± 1.7	0.15 ± 0.21	**771**
4-48	68.4 ± 12.8	28.9 ± 13.1	2.4 ± 0.042	0.31 ± 0.016	**750**
**Allele with mutation in Zn finger**					
66-21	92.1 ± 1.9	7.2 ± 1.8	0.56 ± 0.33	0.16 ± 0.23	**1129**
**Alleles with premature stop codons**					
100-37	1.8 ± 2.0	11.5 ± 3.8	28.2 ± 4.6	58.6 ± 10.3	**630**
114-33	0.24 ± 0.34	4.3 ± 0.99	12.1 ± 7.6	83.4 ± 8.3	**677**
9A9-18	0	0	0	100	**410**
4E2-14	0.36 ± 0.5	2.3 ± 2.2	6.25 ± 2.8	91.1 ± 0.07	**414**
616	0.1 ± 0.14	31.5 ± 7.8	20 ± 1.4	48 ± 9.9	**687**
653	4 ± 0.0	14 ± 4.2	9.5± 3.5	77 ± 14.1	**414**

Dorsal-ventral patterning defects were quantitated by collecting eggs from *spn-E^mutant^/spn-E^∆125^* female flies and determining the percentage of eggs with two dorsal appendages (wild-type), fused dorsal appendages, no dorsal appendages, and eggs that were collapsed. Calculations are from two separate experiments, each consisting of three 24-hr egg collections, with the exception of 653 for which the data were from two separate experiments, each consisting of two 24-hr egg collections.

We found two mutations in amino acids in the DExH box motif, region II (155-55^E239K^, 23-17^H241Q^). Mutation of the Glu in this region leads to a less severe phenotype than the DExH box mutations discussed above, where the majority of eggs laid are collapsed but a larger percentage of eggs with fused or no dorsal appendages are present. Additionally, mutation of the His in the DExH box (23-17^H241Q^) sequence resulted in an even less severe phenotype with 55% of the eggs laid having wild-type patterning.

Females with mutations in SPN-E that lie outside of the predicted functional domains lay eggs with a milder phenotype than those with mutations within the DExH box domain. Mutation of amino acid 508 (9A2-17^P508L^), which changes a conserved Pro to Leu in between the DExH box and the Tudor domains, produced a mild D/V patterning defect with 60% of the eggs laid having a wild-type appearance (Figure 1A, Table 2).

Flies expressing SPN-E with a mutation in the Zn finger motif (66-21^H1422L^) lay eggs with an extremely mild D/V patterning phenotype, resulting in 92% of the eggs having a wild-type appearance.

Interestingly, eggs laid by females from lines 100-37^splicesite^, 616^R1081*^, and 653^Y636*^ show weaker D/V phenotypes than the other mutant *spn-E* alleles that also did not express detectable protein. Line 100-37 has a splice site mutation at base pair 488. If this intron was not spliced properly, then a truncated SPN-E protein should be made. This protein would truncate before the DExH box domain. Because this protein would stop before the peptide to which our antibody was made, we were not able to determine if this shortened SPN-E protein was expressed in 100-37 ovaries. However, when RNA isolated from line 100-37 was analyzed by RT-PCR using primers surrounding the affected intron, the amplified product ran at the same size as wild-type on an agarose gel, indicating that splicing was not altered in 100-37 mutant ovaries (data not shown). We also did not detect protein of the correct size for SPN-E by Western blotting. Nevertheless, the weaker mutant phenotype indicates that at least some functional protein may be made by this allele. Along these same lines, eggs laid by females expressing genetically characterized *spn-E* hypomorphic alleles, *spn-E^616^*, and *spn-E^653^* (Gillespie and Berg 1995) show a similar phenotype to eggs laid by females from the *spn-E^100-37^* allele. We have not been able to detect SPN-E protein from *spn-E^616^* and *spn-E^653^* ovaries, even though our antibody should be capable of recognizing the truncated form of SPN-E that could be made by this allele (Figure 1, Figure S1). It is possible that *spn-E^616^*, *spn-E^653^*, and *spn-E^100-37^* express protein that we are unable to detect by our assay.

To further analyze the D/V phenotype, we examined the expression/localization of the dorsal determinant Gurken (GRK) by immunohistochemistry. At oogenesis stage 8, GRK localizes to the dorsal-anterior corner of the oocyte, where it is necessary to signal to the dorsal follicle cells to establish embryonic dorsal fate. In support of the above data, we found that GRK levels at the dorsal corner of the oocyte are reduced in the *spn-E* mutant ovaries to the same extent as the D/V phenotypes described above, with those alleles that show the most severe D/V patterning defects having the lowest GRK levels (Figure S3). Additionally, we did not detect any Grk expression in the stronger *spn-E* alleles, 2A9^T145I^, 7G2^T233I^, 8D4^S435F^, 114-33^R918*^, 9A9^W632*^, or 4E2^R695*^. For the 155-55^E239K^, 100-37^splicesite488^, 616^R1081*^, and 653^Y636*^ alleles, which have moderate D/V patterning defects, we did not detect significant Grk levels at the dorsal-anterior corner of the oocyte at stage 9 and beyond; however, we did detect reduced GRK in the oocyte at the earlier stages (Figure S3, arrows). Therefore, it is possible that some GRK is expressed and localized at the later oogenesis stages in these alleles, but the level is not detectable by our immunofluorescent assay.

From the above data, it seems that the DExH box domain of SPN-E is quite important for embryonic axis specification. However, the Zn finger seems to be dispensable.

### The DExH box domain is important for SPN-E function in Aubergine nuage localization

**Figure 2:**
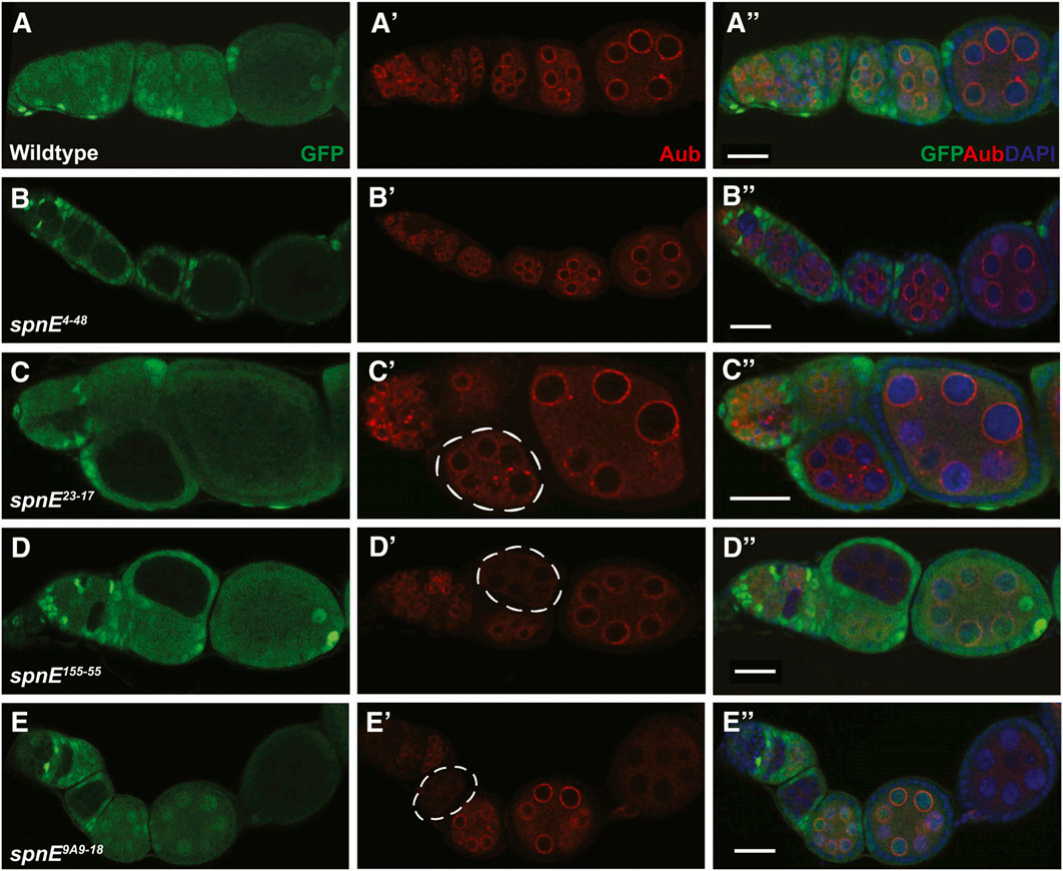
AUB nuage localization is lost in some, but not all, of the *spn-E* mutant egg chambers. *spn-E* mutant germline clones are marked by the absence of GFP. All egg chambers were stained with α-GFP (green), α-AUB (red), and DAPI to mark the DNA. In wild-type egg chambers, AUB localizes around the nurse cell nuclei to a structure known as the nuage (A-A′′). *spn-E^4-48^* (B-B′′) and *spn-E^66-21^* (not shown) show wild-type localization of AUB to the nuage. *spn-E^23-17^* (C-C′′) and *spn-E^9A2-17^* (not shown) show an intermediate phenotype where AUB expression is punctate and only partially localized to the nuage (C′, chamber outlined). In the *spn-E^155-55^* DExH box mutant allele (D-D′′) as well as most of the other DExH box alleles (not shown), AUB is not localized to the nuage and levels of AUB protein appear to be strongly decreased in mutant egg chambers (D′, outlined). This phenotype is also seen in *spn-E^9A9-18^* mutant egg chambers (E-E′′) as well as the remainder of the *spn-E* alleles that do not express detectable protein (not shown). Scale bars = 20 μm.

AUB and SPN-E co-localize to the nuage in wild-type ovaries and AUB localization depends on SPN-E function (Findley *et al.* 2003; Patil and Kai 2010). Therefore, we determined if AUB was localized properly in the different *spn-E* mutant ovaries using immunohistochemistry. To compare mutant and wild-type expression/localization in the same ovary, we generated clones of homozygous mutant egg chambers in an otherwise heterozygous background using the FLP/FRT technique (Chou and Perrimon 1996). Similar to what we saw with the D/V patterning phenotype, we found that AUB localization is variably affected in the different *spn-E* mutant ovaries. We found that AUB is completely delocalized from the nuage in ovaries isolated from *spn-E* mutant flies that show the strongest D/V patterning phenotypes, particularly those alleles with DExH box mutations and the *spn-E* alleles that did not express detectable protein. Additionally, when we compared *spn-E* mutant egg chambers side by side with wild-type egg chambers, it appeared that AUB levels were strongly reduced in these mutant egg chambers (Figure 2, D and E, data not shown). *spn-E* 23-17^H241Q^ and 9A2-17^P508L^ females laid eggs with mid-range D/V patterning phenotypes and in ovaries isolated from these alleles AUB partially localized to the nuage, giving a more punctate localization than wild-type (Figure 2C). In ovaries isolated from the weaker *spn-E* alleles, 66-21^H1422L^ and 4-48^E717K^, AUB was localized to the nuage as in wild-type (Figure 2B). The AUB localization studies shown above were performed by analyzing homozygous *spn-E* mutant clones. To show that the phenotype that we see is due to the mutation in the *spn-E* gene and not a secondary background mutation, we also analyzed AUB localization in hemizygous *spn-E* mutant ovaries using the respective *spn-E* allele *in trans* to the *spn-E* deficiency, Δ125 (Figure S2). We found results similar to those shown above, with the exception of the 4-48^E717K^ allele. The 4-48 hemizygous mutant egg chambers showed a little more punctate AUB localization than the clones; however, AUB was still localized to a certain extent. Therefore, similar to what we saw with D/V patterning, ovaries expressing SPN-E with mutations in the DExH box domain give a similar phenotype to the strongest *spn-E* mutant phenotype, with AUB delocalized from the nuage, demonstrating the importance of the DExH box domain to SPN-E function.

### The DExH box domain of SPN-E is necessary for ovarian dynein-dependent molecular transport

**Figure 3:**
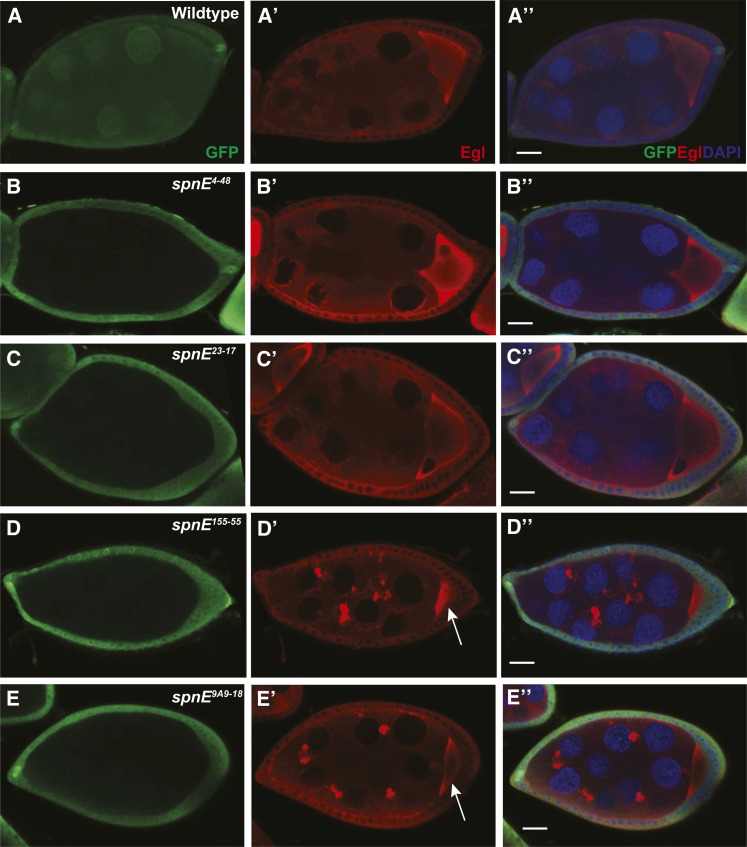
Dynein motor complex aggregates form in some, but not all, *spn-E* mutant ovaries. *spn-E* mutant germline clones are marked by the absence of GFP. All egg chambers were stained with α-GFP (green) to mark clones, α-Egalitarian (EGL) (red), and the DNA dye DAPI. In wild-type egg chambers, EGL is dispersed throughout the nurse cells and localizes to the oocyte (A-A′′). *spn-E^4-48^* (B-B′′), *spn-E^23-17^* (C-C′′), as well as *spn-E^9A2-17^* and *spn-E^66-21^* (not shown) show wild-type EGL localization. In *spn-E^155-55^* DExH box mutant egg chambers (D-D′′), EGL forms aggregates throughout the egg chamber. This phenotype is present in *spn-E^9A9-18^* mutant egg chambers (E-E′′) as well as the DExH box alleles: *spn-E^2A9-14^*, *spn-E^7G2-5^*, *spn-E^8D4-11^*, and the remainder of the *spn-E* alleles that do not express detectable protein (not shown). Note the small size of the oocyte in *spn-E^155-55^* and *spn-E^9A9-18^* egg chambers (arrow in D′ and E′). Scale bars = 20 μm.

In wild-type egg chambers, dynein motor complex proteins are transported from the nurse cells to the oocyte, ultimately leading to a high concentration of protein in the oocyte and a more diffuse pattern in the supporting nurse cells (Figure 3A). This is in contrast to piRNA pathway mutant egg chambers where large aggregates containing components of the dynein motor machinery form in the nurse cells (Navarro *et al.* 2009). These aggregates contain the dynein core motor complex as well as the accessory proteins, Egalitarian (EGL) and Bicaudal-D (BIC-D), and may be sites of retrotransposon sequestration or degradation. We examined ovaries from the different *spn-E* mutants and determined whether dynein aggregates form by immunohistochemistry using EGL as a marker for the aggregates. Similar to what we found with the D/V patterning phenotype, those *spn-E* mutant flies that lay eggs with the most severe D/V patterning defects formed dynein motor complex aggregates (Figure 3, D and E), whereas the *spn-E* mutants that had milder D/V patterning defects did not form ovarian dynein aggregates (Figure 3, B and C). Additionally, in the mutants with the most severe dynein aggregation phenotype, the oocyte failed to grow properly, whereas in the less severe mutants the oocyte appeared to grow normally (Figure 3). The failure of oocyte growth could indicate a failure of the nurse cells to transport their contents into the oocyte, which could result in collapsed eggs. The lack of oocyte growth we found correlates well with the percentage of collapsed eggs laid by the most severe mutants. As above, we confirmed the mutant phenotypes that we saw in homozygous *spn-E* mutant clones in hemizygous ovaries and found the same phenotypes (Figure S3).

### Mutations in the DExH box of SPN-E cause elevated ovarian retrotransposon levels

**Figure 4:**
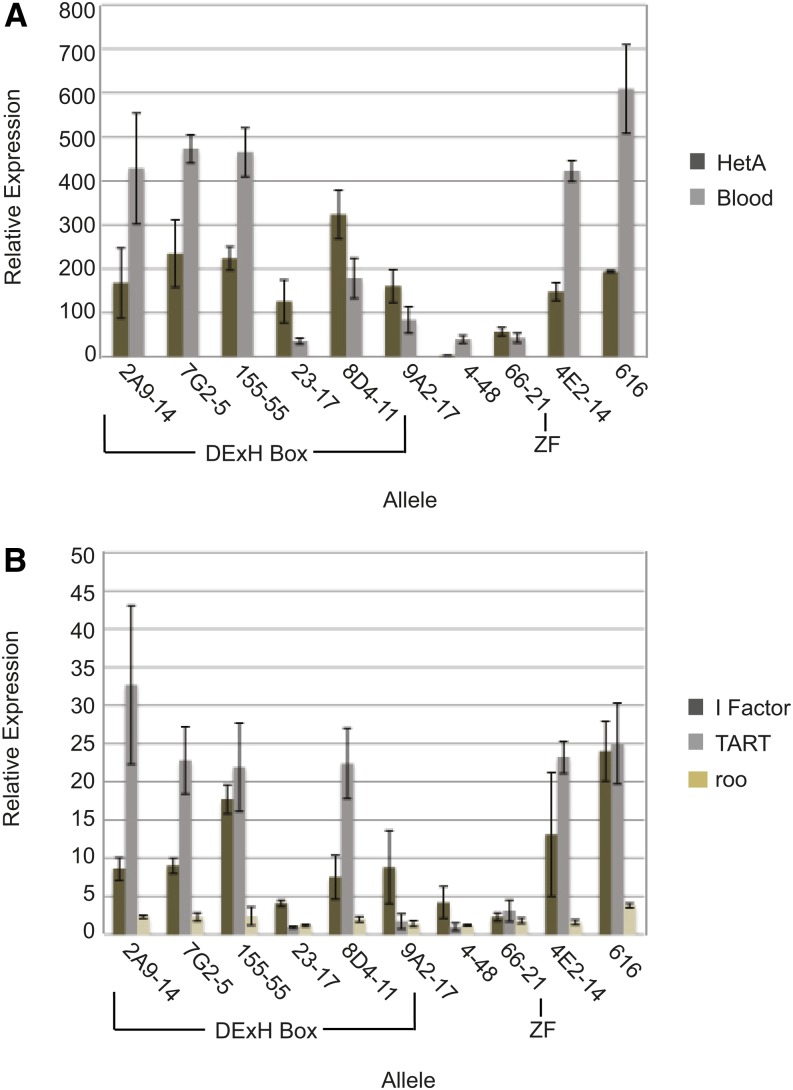
Retrotransposon RNA levels are increased to varying degrees in the various *spn-E* mutant ovaries. (A) Quantitative real-time RT-PCR for the retrotransposons Het-A and Blood using extracts from mutant ovaries (*spn-E^mutant^/spn-E^∆125^*). (B) Quantitative real-time RT-PCR for retrotransposons I Factor, TART, and Roo. Relative expression was calculated in comparison to respective RNA levels obtained from heterozygous siblings for each individual allele. All RNA was normalized to Adh. Error bars represent SD of four experiments using two independent RNA isolates.

Mutations in piRNA pathway proteins result in increased retrotransposon RNA levels in the *Drosophila* ovary (Malone *et al.* 2009; Vagin *et al.* 2006). We measured the RNA level of several germline-specific and one somatic cell-specific retrotransposon in the *spn-E* mutant ovaries using quantitative real-time RT-PCR (Figure 4, Figure S4, Figure S5). The germline-specific retrotransposons included the non-LTR retrotransposons, HetA, TART, and I Factor, and the LTR retrotransposons, Blood and roo, whereas the somatic retrotransposon used was gypsy. We found a similar trend in phenotypic severity for germline retrotransposon RNA expression in the *spn-E* mutant ovaries as we found for the D/V patterning defects, AUB localization, and dynein aggregate formation that we described above. Ovaries from flies expressing SPN-E protein with mutations in the DExH box region had the highest ovarian retrotransposon RNA levels. However, mutations outside of the DExH box region, including the mutation in the Zn finger motif, produced a more moderate phenotype. Interestingly, all alleles showed increased levels of the Blood and HetA retrotransposons to some extent; however, those *spn-E* mutants that gave the strongest developmental phenotypes showed considerably higher levels of HetA and Blood compared with the other alleles. For example, *spn-E^155^*^-55(E239K)^ ovaries had ∼500× the level of Blood and ∼200× the level of HetA retrotransposon RNA as heterozygous controls, whereas *spn-E^66-21(H1422L)^* ovaries had only ∼40× the level of Blood and ∼50× the level of HetA as the heterozygotes. Additionally, only those alleles that gave the strongest developmental phenotypes also had high levels of I Factor, TART, and roo. SPN-E has previously been reported to function only in the ovarian germline cells; therefore, we did not expect to see an effect on the somatic retrotransposon gypsy (Malone *et al.* 2009). However, we did detect a slight increase in gypsy expression in most of the *spn-E* alleles that we examined. The level of expression was significantly lower than that seen in the *dSETDB1* mutant, *egg*. dSETDB1 function is required in both the somatic and germline cells of the ovary; however, its silencing of gypsy transcription has been attributed to its somatic function (Rangan *et al.* 2011). Therefore, it is possible that SPN-E may have a function in silencing retrotransposons in the somatic ovary cells or gypsy may also be expressed in the germline cells of the ovary. In support of our data, Malone *et al.* (2009) showed a slight decrease in gypsy piRNA ping-pong pairs in *spn-E* mutant ovaries. From the above data, it seems likely that the resulting piRNA phenotypes are due to the upregulation of multiple retrotransposons.

As mutations in the DExH box region of SPN-E produce a similar phenotype to the strongest phenotypes ascribed to *spn-E* mutant ovaries in all of the assays we used, our cumulative data point to an important role for the DExH box region in SPN-E function during *Drosophila* oogenesis.

## Discussion

We characterized 14 *spn-E* mutant alleles using multiple phenotypic criteria and identified point mutations in the *spn-E* gene in these alleles. Our results strongly indicate that the DExH box helicase domain of SPN-E is necessary for function, whereas the Zn finger domain seems to be dispensable at least for the functions assessed here.

### The DExH box domain of SPN-E is necessary for function during oogenesis

Our sequencing efforts identified five *spn-E* alleles with mutations in the DExH box domain. Three of the amino acids affected by these mutations have been shown to be important for DExH box helicase activity in other DExH box-containing proteins. These include mutations in two motifs necessary for ATP hydrolysis and binding, motifs I and II. The mutation that we identified at amino acid 145 within motif I changes a Thr, a polar uncharged amino acid, to an Ile, a nonpolar hydrophobic amino acid. This amino acid seems to be critical for SPN-E function as mutant ovaries display a severe oogenesis phenotype. Previous mutational studies of vaccinia virus nucleoside triphosphate phosphohydrolase I (NPH-I) showed that an amino acid with –OH group at the analogous position in the NPH-I DExH box is necessary for its ATPase activity (Martins *et al.* 1999). For example, when Thr62 of NPH-I is changed to an Ala or Val, both of which are nonpolar hydrophobic amino acids, the ATPase activity of NPH-I is decreased dramatically. However, when Thr62 is changed to Ser, an uncharged polar amino acid with –OH group, ATPase activity was not affected. This finding is also supported by mutational analysis of the yeast splicing factor, Prp16, where expression of the protein with a change of Thr380 to either Ala or Val leads to lethality, whereas a change of Thr380 to Ser does not affect yeast growth (Hotz and Schwer 1998).

Less severe phenotypes resulted from mutations in the DExH box motif itself. Mutation of Glu239 to Lys resulted in a moderate phenotype, whereas mutation of His241 to Gln resulted in an even milder phenotype. Mutational analysis of Glu140 within the NPH-I DExH box showed that an acidic side chain at this position is necessary for ATP hydrolysis (Martins *et al.* 1999). This is also true for the yeast splicing factor, Prp16 (Hotz and Schwer 1998). Our analysis agrees with this conclusion as the change we identified in SPN-E is a substitution of Glu, an acidic amino acid to Lys, a polar basic amino acid. The mutation at amino acid 241 results in a change from His to Gln. His and Gln are partially isosteric, which may account for the less severe phenotype. Mutational analysis of NPH-I also showed a similar result where a His to Gln change decreased ATPase activity to 42%, whereas a more dramatic effect was seen when His was changed to Ala or Asp. Again, this is also in agreement with changes in the comparable amino acid of Prp16 (Hotz and Schwer 1998; Martins *et al.* 1999).

We also have identified mutations in two uncharacterized amino acids within the DExH box domain. This includes a Ser-to-Phe change at amino acid 435 in motif V, which has been implicated in RNA binding (Rocak and Linder 2004). This mutation gave one of the strongest phenotypes, indicating that this amino acid is critical for SPN-E function and is consistent with the proposal that SPN-E interacts with RNA.

Our results indicate that the DExH box domain of SPN-E is necessary for retrotransposon silencing and oocyte patterning. Given that DExH box domains have helicase activity and work to change nucleic acid conformation, how could the DExH box in SPN-E contribute to its function in piRNA biogenesis? Previous reports showed that in *spn-E* mutant ovaries the levels of all piRNAs are depleted and that piRNA ping-pong piRNAs are not generated (Malone *et al.* 2009). Therefore, it is possible that SPN-E could function to change piRNA structure to make the RNA more accessible to other piRNA pathway proteins, such as nucleases or chaperones at one or multiple points in the biogenesis pathway. This could include binding to and unwinding: pre-piRNAs for primary piRNA processing to occur: secondary piRNAs for incorporation into the silencing/cleavage complex; or retrotransposon RNAs for cleavage by Aub or Ago3. Alternatively, SPN-E could function indirectly in piRNA biogenesis by affecting the translation of a key piRNA pathway protein such as AUB. Given that AUB levels are reduced in *spn-E* mutant egg chambers, it seems that SPN-E function may be necessary to maintain AUB protein levels. Therefore, it is possible that SPN-E functions in AUB translation. However, it is just as likely that SPN-E function may be necessary for AUB stabilization. Given that AUB binds to piRNAs it is possible that without piRNAs, AUB may become unstable. Therefore, because piRNA levels are low in *spn-E* mutant ovaries, AUB may be degraded due to lack of piRNA binding. Alternatively, SPN-E may associate with AUB either in conjunction with or after piRNA processing. Without piRNA generation, SPN-E and AUB may not form a complex leading to AUB destabilization.

Interestingly, mutation of amino acid 1422, which is located in the putative Zn finger, produced a very mild phenotype with 92% of eggs laid being wild-type. The mutation that we identified is in an amino acid crucial for Zn coordination, indicating that this mutation would most likely disrupt Zn finger activity (Pabo *et al.* 2001). The Zn finger seems to be unique to *Drosophila* because we did not find a Zn finger in SPN-E homologs such as TDRD9 from humans, mouse, and zebrafish using the protein sequences deposited in the Ensemble Genome Browser release 73 (Figure 1A). These results indicate that Zn finger activity may not be important for SPN-E function in embryonic patterning.

### SPN-E may have functions independent of piRNA biogenesis

We found that some *spn-E* alleles cause elevated levels of all of the retrotransposons that we examined, whereas others caused elevated levels of only HetA and/or Blood. Blood and HetA seem to be the most sensitive elements to piRNA pathway perturbation (Lim and Kai 2007; Sienski *et al.* 2012; Vagin *et al.* 2006). This could be due to their placement within the piRNA clusters or, perhaps, differential sensitivity of the clusters themselves to piRNA pathway perturbations. The alleles that caused only a mild elevation of a subset of retrotransposon RNA consistently produced weaker developmental phenotypes. It is possible that the higher levels of retrotransposon RNA that we found in the weaker alleles may be due to decreased SPN-E protein levels and that retrotransposon silencing is a more sensitive readout for changes in SPN-E function than the developmental phenotypes that we examined. For the most part, there is good correlation between elevated ovarian retrotransposon levels and the severity of the *spn-E* phenotype, with those alleles that have high levels of all retrotransposons showing the strongest phenotypes.

The one exception to the above statement is *spn-E^4-48^*. *spn-E^4-48^* ovaries only show a slight increase in retrotransposon levels, yet the eggs laid by these females have a moderate D/V phenotype. Most piRNA pathway mutant ovaries have an active Chk-2-dependent DNA damage checkpoint (Klattenhoff *et al.* 2007; Pane *et al.* 2007). It is thought that checkpoint activation is due to the massive amounts of DNA double strand breaks that occur from elevated retrotransposition in these ovaries. However, this has not been directly shown. It is known that activation of the checkpoint leads to embryonic patterning defects for most piRNA pathway mutants (Klattenhoff *et al.* 2007; Pane *et al.* 2007). This does not seem to be the case for *spn-E*, however, because the D/V patterning defects of *spn-E* mutants are not suppressible by checkpoint inhibition (Pane *et al.* 2007). Therefore, SPN-E probably has functions in addition to its function in piRNA biogenesis. One of these may be to control cytoplasmic streaming, as premature cytoplasmic streaming has been reported for certain *spn-E* mutant alleles (Martin *et al.* 2003). *orb* (oo18 RNA binding protein), a Cytoplasmic Polyadenylation Element binding protein that functions in translational regulation, mutant ovaries also show premature cytoplasmic streaming similar to what is seen in *spn-E* mutant egg chambers (Martin *et al.* 2003). The streaming defects could cause *grk* RNA mislocalization that, in turn, would lead to embryonic patterning defects. Orb levels are reduced in *spn-E* mutant ovaries; therefore, it is possible that Orb may be either a direct or an indirect target of SPN-E function (Martin *et al.* 2003).

Additionally, it has been shown that in *I-R* hybrid dysgenic crosses where the only retrotransposon that is upregulated is I Factor, the DNA damage checkpoint is not activated, yet moderate D/V patterning defects are seen in the eggs laid by the *I-R* dysgenic females (Orsi *et al.* 2010). The D/V patterning phenotype in IR dysgenic eggs is similar to what we saw for *spn-E^4-48^* eggs. Additionally, the retrotransposon that is most affected in *spn-E^4-48^* ovaries is I Factor. The D/V defects that arise in eggs laid by dysgenic females are thought to arise from a competition of I Factor and *grk* RNA for access to the microtubule motor machinery, which leads to the displacement of *grk* RNA and the resulting D/V patterning defects (Van De Bor *et al.* 2005). It will be interesting to determine whether the DNA double strand break checkpoint is activated in *spn-E^4-48^* ovaries and why I Factor levels are the most impacted by the *spn-E^4-48^* mutation.

In addition to D/V patterning and checkpoint activation, it seems that dynein aggregate formation is also sensitive to retrotransposon levels. We found that in *spn-E* mutants that have only a slight increase in retrotransposon levels, dynein aggregates do not form. This is also true in *I-R* dysgenic ovaries (Orsi *et al.* 2010). Dynein aggregate formation is due to activation of the Chk-2 checkpoint in *spn-E* mutant ovaries (Navarro *et al.* 2009). Therefore, dynein aggregate formation, as well as checkpoint activation, is sensitive to ovarian retrotransposon levels.

Our experiments have identified several mutations in the DExH box helicase domain of SPN-E implicating helicase function as important for SPN-E function, especially in the piRNA pathway. Although these mutations could affect protein folding, we think that the mutations we have identified affect protein function rather than protein structure because the ovarian protein levels for several of these new alleles were close to wild-type. It is interesting to note that we did not obtain mutations in the highly conserved Tudor domain. It is possible that mutations in the Tudor domain could render SPN-E unstable. However, it is curious that the 616^R1081*^ allele has a mutation that causes a premature stop after the Tudor domain, whereas the rest of the alleles with premature stop codons would cause protein truncation before the Tudor domain. This allele has a weaker D/V patterning phenotype than these other alleles, indicating that if this allele does produce protein, then the Tudor domain may be an important functional domain of SPN-E. A more directed mutagenesis approach may be necessary to determine the relevance of the Tudor domain for SPN-E function.

## Supplementary Material

Supporting Information
