# Supplementary material for: The DExH Box Helicase Domain of Spindle-E Is Necessary for Retrotransposon Silencing and Axial Patterning During Drosophila Oogenesis
Source: G3 (Bethesda). 2014 Sep 19;4(11):2247–57. doi: 10.1534/g3.114.014332 (PMC4232550; doi:10.1534/g3.114.014332)
Supplement: Supporting Information [file supp_4_11_2247__index.html]

The DExH Box Helicase Domain of Spindle-E Is Necessary for Retrotransposon Silencing and Axial Patterning During Drosophila Oogenesis — Supporting Information 

# The DExH Box Helicase Domain of Spindle-E Is Necessary for Retrotransposon Silencing and Axial Patterning During *Drosophila* Oogenesis

## Supporting Information for Ott, Nguyen, and Navarro, 2014

**Files in this Data Supplement:**

- Supporting Information - Figures S1-S5 (PDF, 584 KB)
- Figure S1 - No detectable protein is made from *spn-E* alleles that have premature stop codon mutations. (PDF, 152 KB)
- Figure S2 - Similar to homozygous *spn-E* mutant egg chambers, AUB nuage localization is lost in some, but not all of the *spn-E* hemizygous mutant egg chambers. (PDF, 378 KB)
- Figure S3 - Similar to homozygous *spn-E* mutant egg chambers, dynein motor complex aggregates form and Gurken is not properly localized in some *spn-E* hemizygous mutant ovaries. (PDF, 235 KB)
- Figure S4 - Gypsy retrotransposon levels are slightly elevated in some of the *spn-E* mutant ovaries. (PDF, 128 KB)
- Figure S5 - Het-A, IF and Blood retrotransposon levels are elevated in *spn-E653/spn-EΔ125* mutant ovaries. (PDF, 138 KB)
