# Supplementary material for: The DExH Box Helicase Domain of Spindle-E Is Necessary for Retrotransposon Silencing and Axial Patterning During Drosophila Oogenesis
Source: G3 (Bethesda). 2014 Sep 19;4(11):2247–57. doi: 10.1534/g3.114.014332 (PMC4232550; doi:10.1534/g3.114.014332)
Supplement: Supporting Information [file supp_g3.114.014332_014332SI.pdf]

G3·Genes | Genomes | Genetics

The DExH box helicase domain of Spindle-E is necessary for retrotransposon silencing and axial patterning during Drosophila oogenesis

Kristen M. Ott [†,*], Tram Nguyen[†] and Caryn Navarro [†,*,‡]

[†] Department of Medicine
Biomedical Genetics
Boston University School of Medicine
Boston, MA 02118

[*] Graduate Program in Genetics and Genomics
Boston University School of Medicine
Boston, MA 02118

[‡] Corresponding author:

Caryn Navarro
72 E. Concord Street, E222
Boston University School of Medicine
Boston, MA 02118

Phone: (617) 638-4278
Email: cnavarro@bu.edu

DOI: 10.1534/g3.114.014332

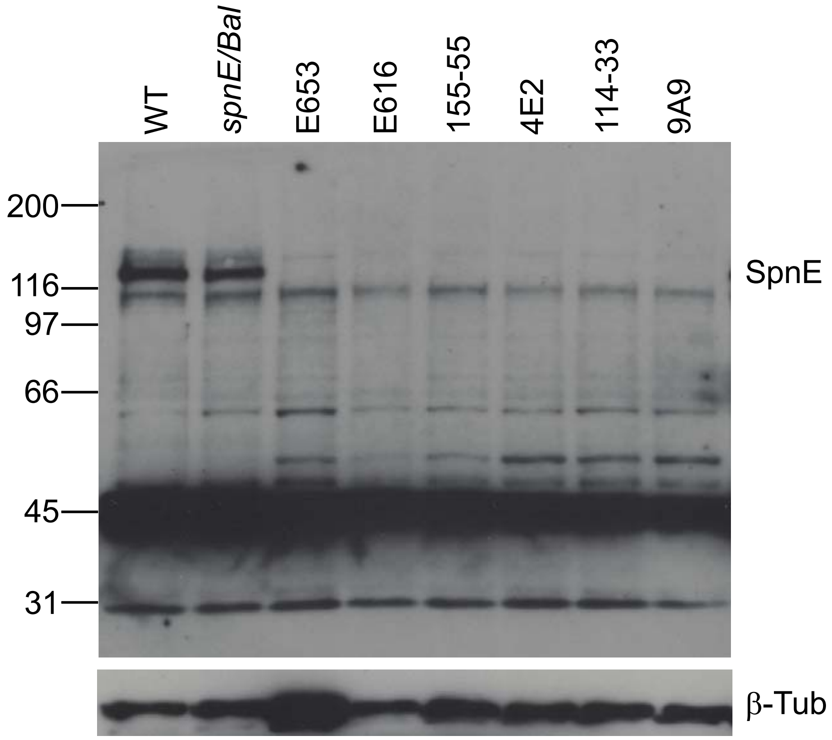

Figure S1 No detectable protein is made from *spn-E* alleles that have premature stop codon mutations. Protein was isolated from hemizygous ovaries of the genotype *spn-E*^{mutant}/*spn-E*$^{\Delta 125}$. The gel for this western blot was run for a shorter amount of time than the one shown in Figure 1, in order to keep any truncated protein that might be expressed on the gel. Additionally this blot was overexposed to try to detect any protein that may not be expressed highly. The heavy band that runs at the 45kDa marker represents yolk protein that reacts highly with the IgG present in the serum.

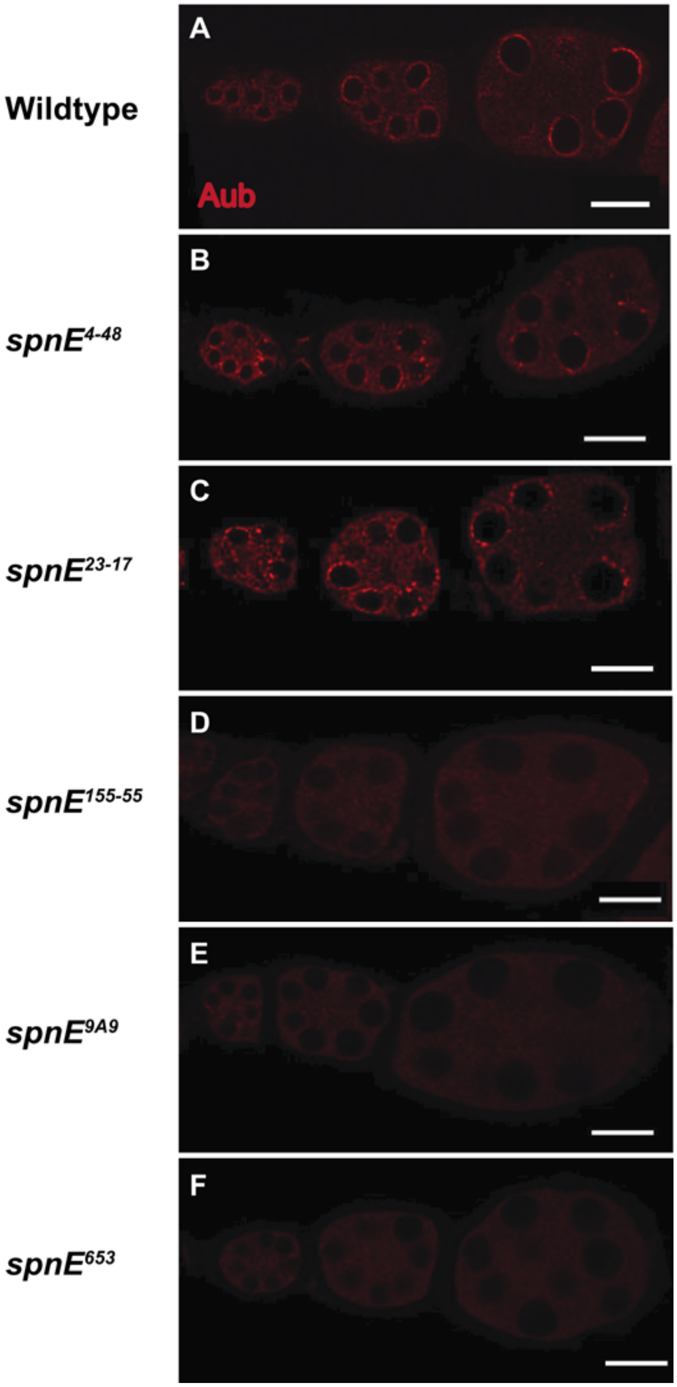

Figure S2 Similar to homozygous *spn-E* mutant egg chambers, AUB nuage localization is lost in some, but not all of the *spn-E* hemizygous mutant egg chambers. Hemizygous mutant ovaries of the genotype *spn-*E^{mutant}/*spn-*$E^{\Delta 125}$ were stained with α-AUB (red) to assess AUB nuage localization. (**A**) Wildtype ovaries of the genotype *spn-*$E^{\Delta 125}$/*Balancer* show AUB localization around the nurse cell nuclei to the nuage. (**B**) *spn-*$E^{4\text{-}48}$ and *spn-*$E^{23\text{-}17}$ mutant ovaries, which represent the class of weaker *spn-E* alleles, show a partial localization of AUB to the nuage. For the 4-48 allele this is slightly different from what is shown in Figure 2 when the AUB localization phenotype was determined in *spn-E* homozygous mutant egg chambers. (**D, E, F**) In the *spn-E* alleles, 155-55, 9A9 and 653, AUB is not localized to the nuage and the levels of AUB protein in the ovary seem to be reduced. This is consistent with what is shown in Figure 2. Scale bars = 20μm.

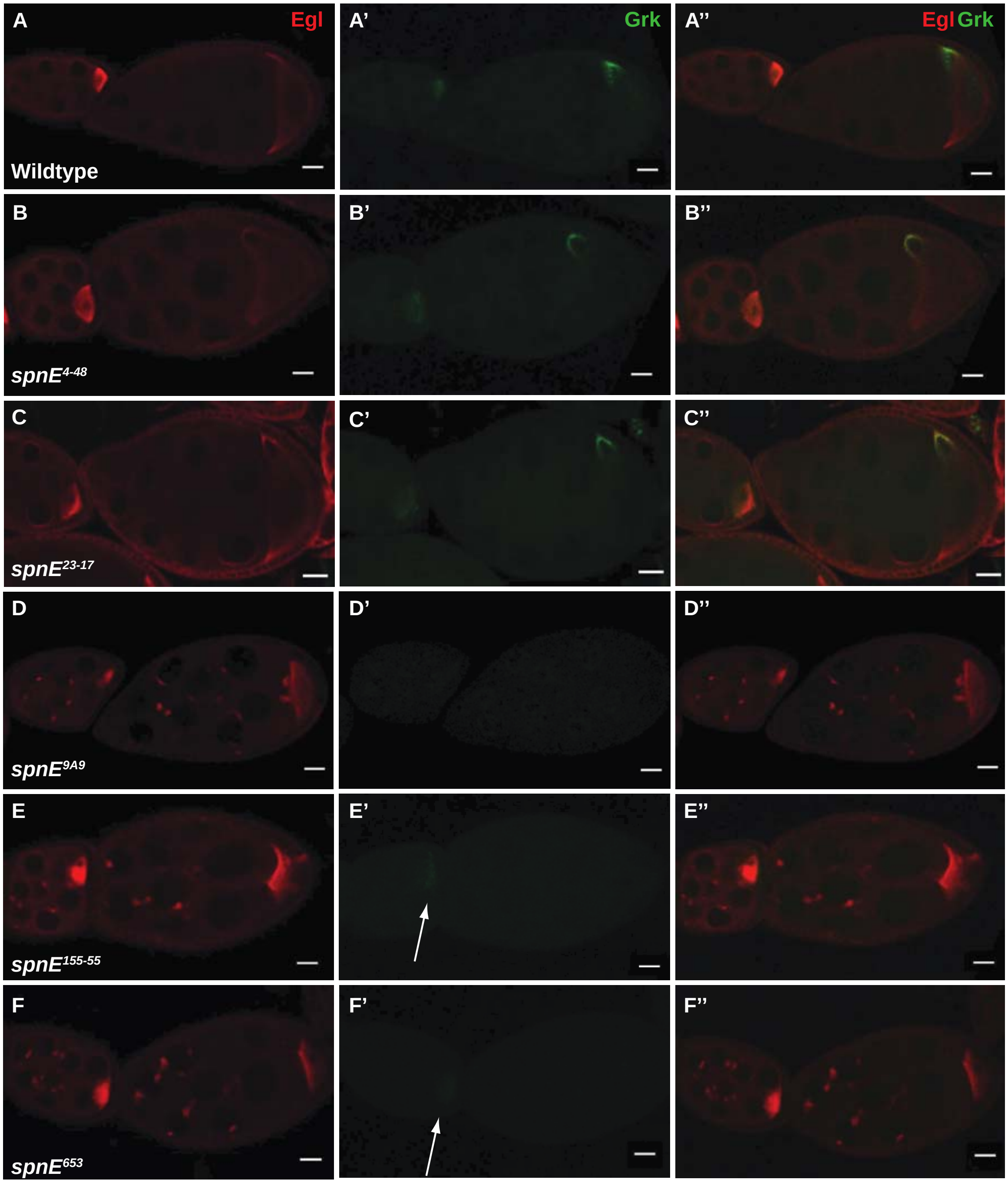

Figure S3 Similar to homozygous *spn-E* mutant egg chambers, dynein motor complex aggregates form and Gurken is not properly localized in some *spn-E* hemizygous mutant ovaries. *spn-E*^{mutant}/*spn-E*$^{\Delta 125}$ were stained with α-EGL (**A-F,** Red) to visualize the dynein motor complex and α-GRK (**A'-F',** Green). (**A**) In wildtype (*spn-E*$^{\Delta 125}$*/Balancer*) EGL localizes to and within the oocyte (**A)** and GRK forms a tight cresent at the dorsal-anterior corner of a stage 8-9 oocyte (**B**). (Egg chamber to the right of each panel). (**B,C**) In *spn-E*$^{4\text{-}48}$ and *spn-E*$^{23\text{-}17}$ no dynein aggregates form (**B,C**) and GRK is localized similar to wildtype (**B',C'**). These alleles represent the typical phenotype of the weaker *spn-E* alleles. (**D-F**) In *spn-E*^{9A9}, *spn-E*$^{155\text{-}55}$, and *spn-E*^{653} dynein motor aggregates form (**D-F**) and GRK is not localized to the dorsal-anterior corner of the oocyte (**D'-F'**). These alleles represent the typical phenotype of the strong *spn-E* alleles. In the earlier chambers of *spn-E*$^{155\text{-}55}$ and *spn-E*^{653} weak GRK expression is found within the oocyte (**E',F', arrow**).

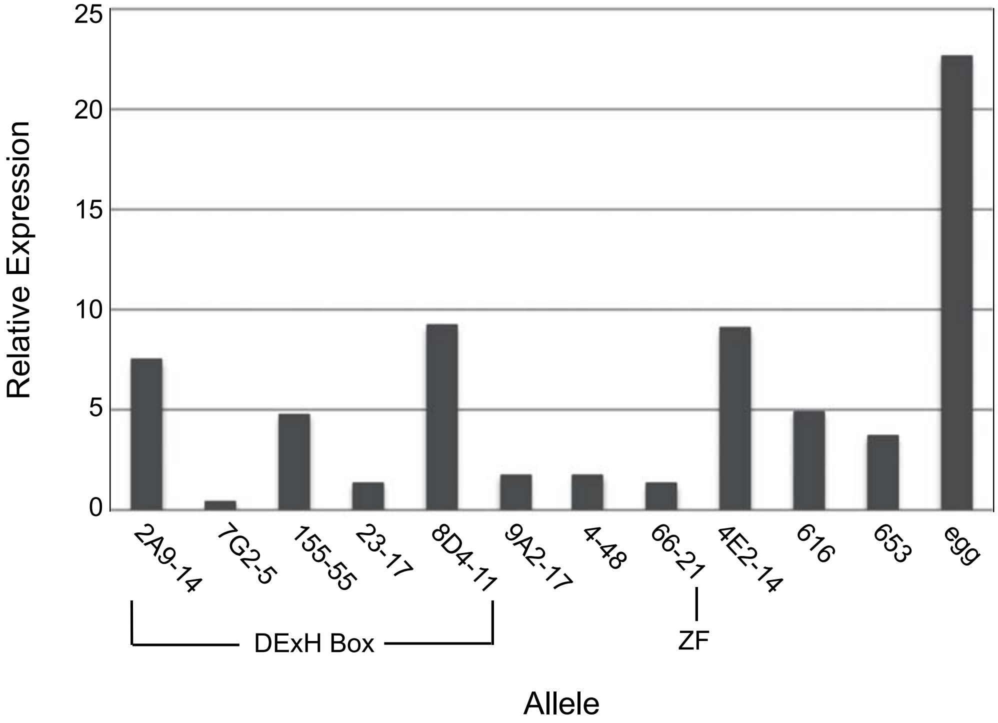

Figure S4 Gypsy retrotransposon levels are slightly elevated in some of the *spn-E* mutant ovaries. Quantitative real time RT-PCR for the gypsy retrotransposon. Relative expression was calculated in comparison to respective RNA levels obtained from heterozygous siblings for each individual allele. All RNA was normalized to Adh.

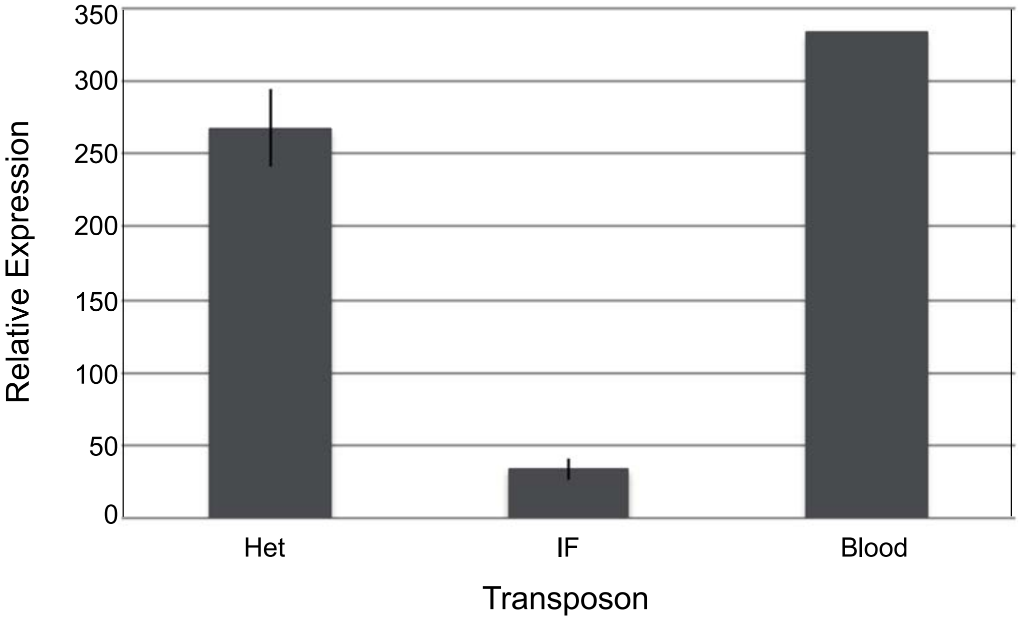

Figure S5 Het-A, IF and Blood retrotransposon levels are elevated in *spn-E*^{653}/ *spn-E*$^{\Delta 125}$ mutant ovaries. Quantitative real time RT-PCR for Het-A, IF and Blood retrotransposons. Relative expression was calculated in comparison to RNA levels obtained from heterozygous siblings for each individual allele. All RNA was normalized to Adh. Error bars represent standard deviation of two independent real time RT-PCR runs.
