# Supplementary material for: The DExH Box Helicase Domain of Spindle-E Is Necessary for Retrotransposon Silencing and Axial Patterning During Drosophila Oogenesis
Source: G3 (Bethesda). 2014 Sep 19;4(11):2247–57. doi: 10.1534/g3.114.014332 (PMC4232550; doi:10.1534/g3.114.014332)
Supplement: Supporting Information [file supp_g3.114.014332_FigureS1.pdf]

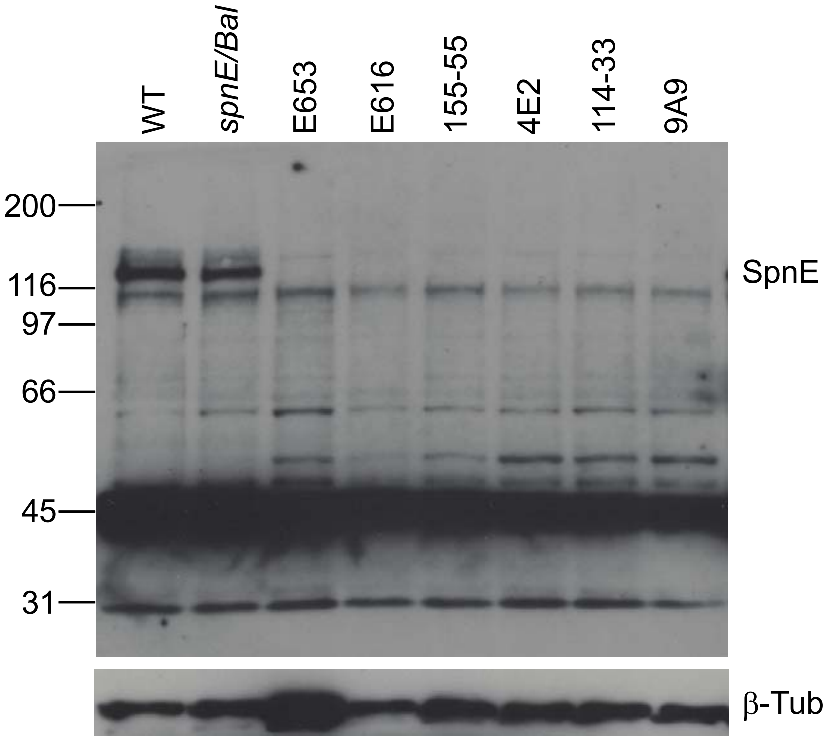


**Figure S1** No detectable protein is made from *spn-E* alleles that have premature stop codon mutations. Protein was isolated from hemizygous ovaries of the genotype $spn\text{-}E^{mutant}/spn\text{-}E^{\Delta 125}$. The gel for this western blot was run for a shorter amount of time than the one shown in Figure 1, in order to keep any truncated protein that might be expressed on the gel. Additionally this blot was overexposed to try to detect any protein that may not be expressed highly. The heavy band that runs at the 45kDa marker represents yolk protein that reacts highly with the IgG present in the serum.
