# Supplementary material for: The DExH Box Helicase Domain of Spindle-E Is Necessary for Retrotransposon Silencing and Axial Patterning During Drosophila Oogenesis
Source: G3 (Bethesda). 2014 Sep 19;4(11):2247–57. doi: 10.1534/g3.114.014332 (PMC4232550; doi:10.1534/g3.114.014332)
Supplement: Supporting Information [file supp_g3.114.014332_FigureS2.pdf]

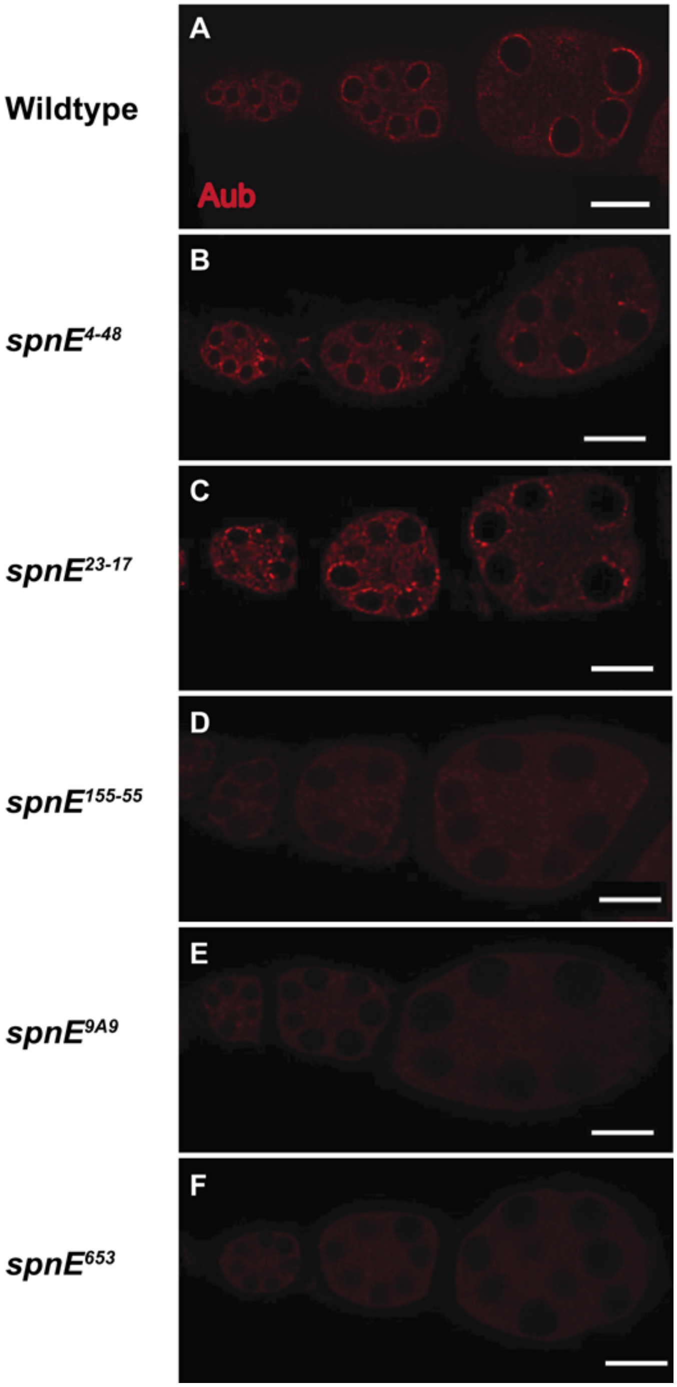

Figure S2 Similar to homozygous *spn-E* mutant egg chambers, AUB nuage localization is lost in some, but not all of the *spn-E* hemizygous mutant egg chambers. Hemizygous mutant ovaries of the genotype *spn-E*^{mutant}/*spn-E*$^{\Delta 125}$ were stained with α-AUB (red) to assess AUB nuage localization. (**A**) Wildtype ovaries of the genotype *spn-E*$^{\Delta 125}$/*Balancer* show AUB localization around the nurse cell nuclei to the nuage. (**B**) *spn-E*$^{4\text{-}48}$ and *spn-E*$^{23\text{-}17}$ mutant ovaries, which represent the class of weaker *spn-E* alleles, show a partial localization of AUB to the nuage. For the 4-48 allele this is slightly different from what is shown in Figure 2 when the AUB localization phenotype was determined in *spn-E* homozygous mutant egg chambers. (**D, E, F**) In the *spn-E* alleles, 155-55, 9A9 and 653, AUB is not localized to the nuage and the levels of AUB protein in the ovary seem to be reduced. This is consistent with what is shown in Figure 2. Scale bars = 20μm.
