# Supplementary material for: The DExH Box Helicase Domain of Spindle-E Is Necessary for Retrotransposon Silencing and Axial Patterning During Drosophila Oogenesis
Source: G3 (Bethesda). 2014 Sep 19;4(11):2247–57. doi: 10.1534/g3.114.014332 (PMC4232550; doi:10.1534/g3.114.014332)
Supplement: Supporting Information [file supp_g3.114.014332_FigureS3.pdf]

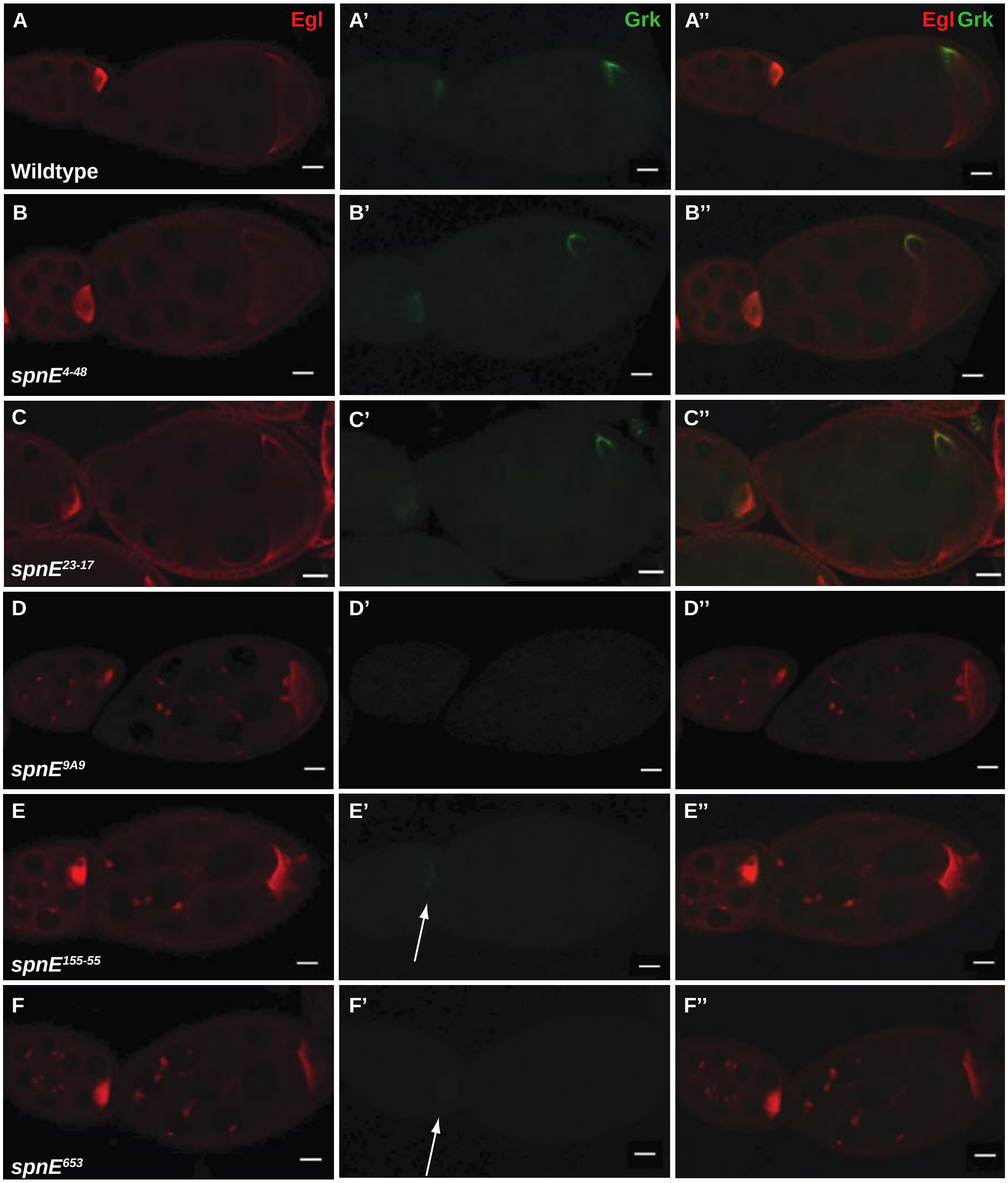

Figure S3 Similar to homozygous *spn-E* mutant egg chambers, dynein motor complex aggregates form and Gurken is not properly localized in some *spn-E* hemizygous mutant ovaries. *spn-E^{mutant}/spn-E$^{\Delta125}$* were stained with α-EGL (**A-F,** Red) to visualize the dynein motor complex and α-GRK (**A'-F',** Green). (**A**) In wildtype (*spn-E$^{\Delta125}$/Balancer*) EGL localizes to and within the oocyte (**A)** and GRK forms a tight cresent at the dorsal-anterior corner of a stage 8-9 oocyte (**B**). (Egg chamber to the right of each panel). (**B,C**) In *spn-E$^{4\text{-}48}$* and *spn-E$^{23\text{-}17}$* no dynein aggregates form (**B,C**) and GRK is localized similar to wildtype (**B',C'**). These alleles represent the typical phenotype of the weaker *spn-E* alleles. (**D-F**) In *spn-E^{9A9}*, *spn-E$^{155\text{-}55}$*, and *spn-E^{653}* dynein motor aggregates form (**D-F**) and GRK is not localized to the dorsal-anterior corner of the oocyte (**D'-F'**). These alleles represent the typical phenotype of the strong *spn-E* alleles. In the earlier chambers of *spn-E$^{155\text{-}55}$* and *spn-E^{653}* weak GRK expression is found within the oocyte (**E',F', arrow**).
