# Supplementary material for: The DExH Box Helicase Domain of Spindle-E Is Necessary for Retrotransposon Silencing and Axial Patterning During Drosophila Oogenesis
Source: G3 (Bethesda). 2014 Sep 19;4(11):2247–57. doi: 10.1534/g3.114.014332 (PMC4232550; doi:10.1534/g3.114.014332)
Supplement: Supporting Information [file supp_g3.114.014332_FigureS4.pdf]

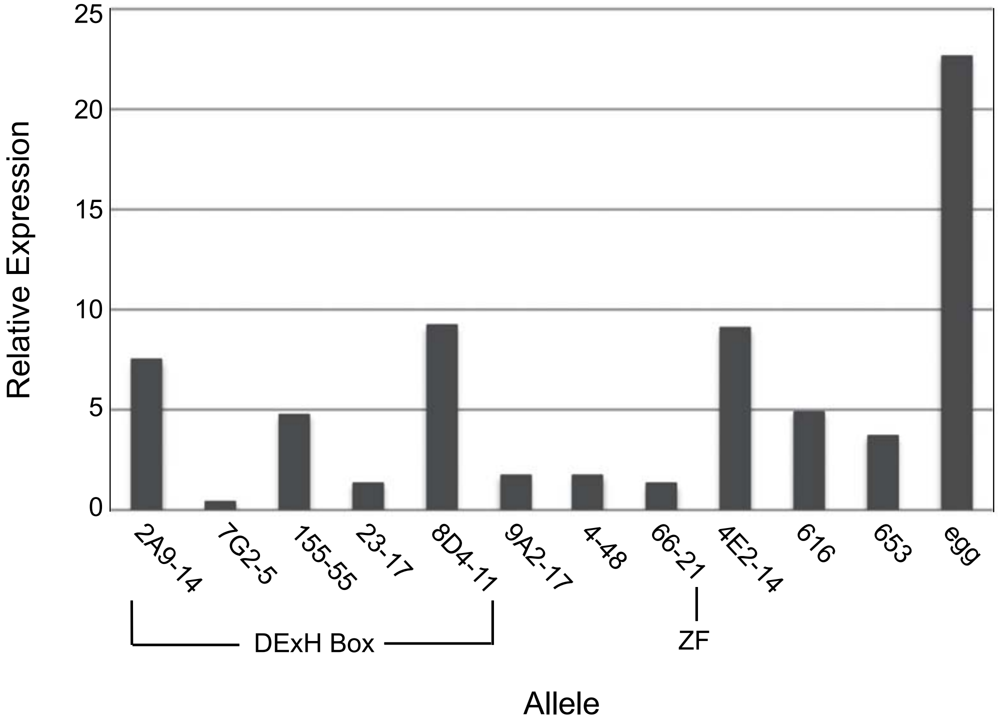


**Figure S4** Gypsy retrotransposon levels are slightly elevated in some of the *spn-E* mutant ovaries. Quantitative real time RT-PCR for the gypsy retrotransposon. Relative expression was calculated in comparison to respective RNA levels obtained from heterozygous siblings for each individual allele. All RNA was normalized to Adh.
