# Supplementary material for: The DExH Box Helicase Domain of Spindle-E Is Necessary for Retrotransposon Silencing and Axial Patterning During Drosophila Oogenesis
Source: G3 (Bethesda). 2014 Sep 19;4(11):2247–57. doi: 10.1534/g3.114.014332 (PMC4232550; doi:10.1534/g3.114.014332)
Supplement: Supporting Information [file supp_g3.114.014332_FigureS5.pdf]

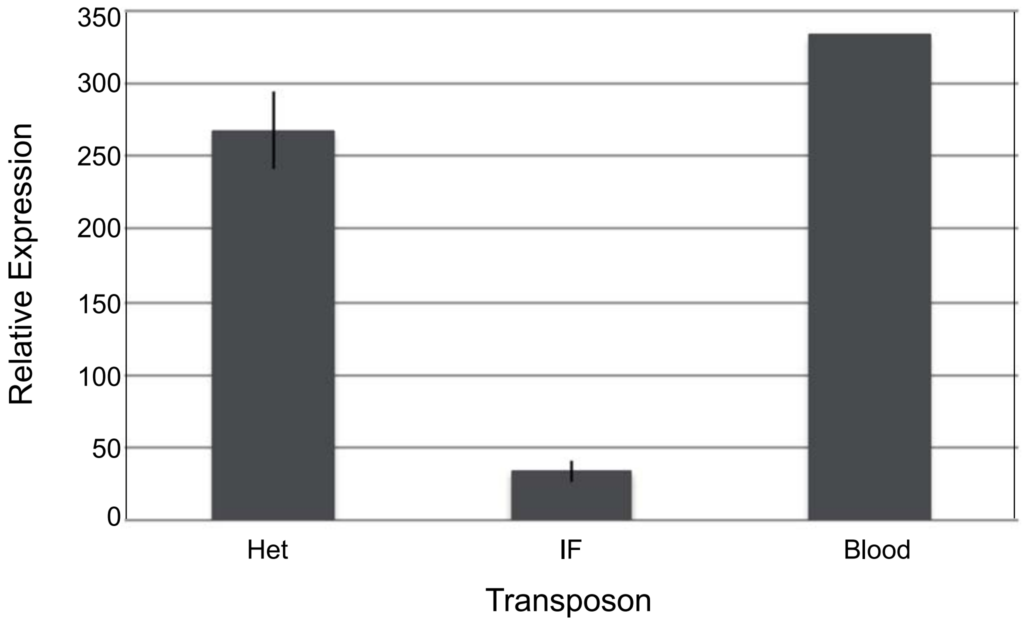

Figure S5 Het-A, IF and Blood retrotransposon levels are elevated in *spn-E*^{653}/ *spn-E*$^{\Delta 125}$ mutant ovaries. Quantitative real time RT-PCR for Het-A, IF and Blood retrotransposons. Relative expression was calculated in comparison to RNA levels obtained from heterozygous siblings for each individual allele. All RNA was normalized to Adh. Error bars represent standard deviation of two independent real time RT-PCR runs.
